# A Japanese Case of Food Protein-induced Enterocolitis Syndrome Caused by Multiple Seafoods

**DOI:** 10.24546/010049096

**Published:** 2024-08-05

**Authors:** TAIKI SATOU, SAEKO YAMAMURA, AKARI TAKAHASHI, SEIICHI TAWAZA, KYOKO OZAWA, YOSHIKO TANAKA, HIROSHI ASADA

**Affiliations:** 1 Department of Pediatrics, Japanese Red Cross Sendai Hospital, Sendai, Japan

**Keywords:** Food protein-induced enterocolitis syndrome, Thymus and activation-regulated chemokine, Oral food challenge, Fish allergy

## Abstract

Food protein-induced enterocolitis syndrome (FPIES) caused by fish and others is prevalent in the Mediterranean regions but is less frequently reported in Japan. This case report describes a 3-year-old Japanese girl who developed FPIES triggered by multiple seafoods, including swordfish, cod, and squid. The diagnosis was confirmed through oral food challenge tests (OFC), which led to repeated vomiting and an increase in thymus and activation-regulated chemokine (TARC) levels. This case highlights the importance of considering fish-induced FPIES in the differential diagnosis of recurrent vomiting in children and suggests the potential utility of TARC levels in diagnosing and monitoring FPIES.

## INTRODUCTION

Food protein-induced enterocolitis syndrome (FPIES) usually occurs in infants and young children and is characterized by recurrent vomiting 1–4 h after ingestion of the causative food. Fish and other seafoods are one of the most predominant causes of FPIES in Mediterranean regions, including Greece, Italy, Spain, and Turkey (1). Some pediatric cases of fish FPIES have been reported in Japan (2, 3), but the exact disease epidemiology remains unknown. Additionally, we found no report of detailed evaluation and clinical diagnosis of FPIES in multiple fish and shellfish. Herein, we report a patient with cod-, swordfish-, and squid-related FPIES, who experienced vomiting after ingesting those food triggers. We used a food challenge test to assess each food trigger.

## CLINICAL CASE

A 3-year-and-3-month-old Japanese girl was referred to our outpatient hospital due to vomiting 3 h after ingesting baked cod with mayonnaise. The patient experienced a similar episode with salted cod a month ago. No skin or respiratory symptoms were observed. She required no medical attention, and all symptoms improved a day after each episode. She experienced four episodes of vomiting after ingesting swordfish at 2 years and 4 months of age. Oral food challenge (OFC) of 100 g of baked swordfish was performed 7 months after these episodes and caused four urticaria lesions in one area 30 min after and frequent vomiting 2 h after ingestion. The patient was admitted to an emergency hospital at 3 years and 2 months of age and received supplemental fluid for vomiting more than 10 times 2 h after ingesting squid. The patient did not experience pallor, or diarrhea during each episode. The patient did not experience symptoms during the first ingestion of cod, swordfish, or squid in her life. The patient’s guardian was instructed to eliminate swordfish and squid. The medical history of the patient indicated multiple urticaria episodes after bathing at 2 years of age. She had no other food allergies, atopic dermatitis, asthma, developmental problems, or a family history of FPIES.

**Figure 1:**
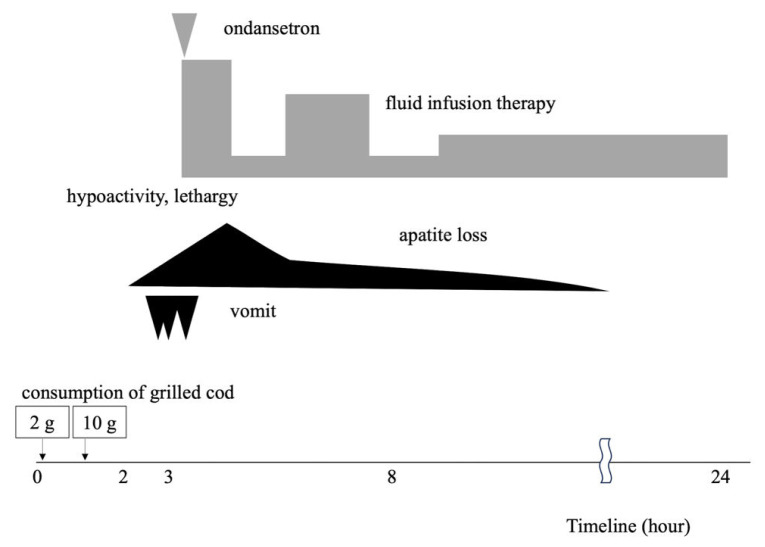
Time course of cod oral food challenge

The following tests were conducted due to the suspicion of fish and mollusk allergy: eosinophil count (166/μl), nonspecific immunoglobulin E (IgE, 105 IU/mL), cod specific IgE (sIgE, 0.15 UA/mL), squid sIgE (0.1 UA/mL), Anisakis sIgE (0.1 UA/mL), thymus and activation-regulated chemokine (TARC, 422 pg/mL), and prick test with raw swordfish (positive, mean wheal diameter 8 mm), raw cod (negative, 2 mm), and raw squid (positive, 6 mm). Additionally, the swordfish antigen-specific lymphocyte stimulation test (ALST) revealed maximum selectivity index of 2.5. Table I shows the previous laboratory results. OFC with 10 g of grilled cod was performed 12 months after the final episode with cod. The patient ingested 2 g of baked cod, followed by 8 g 1 h later, after hospitalization. The patient became hypoactive approximately 2 h after the first ingestion, and pallor, lethargy, and frequent vomiting occurred 1 h later. The patient received supplemental fluids and ondansetron intravenously. Anorexia and hypoactivity lasted for 8 h. Hypotension, hypothermia, diarrhea, or rash including eczema were not observed during OFC. Blood tests were conducted twice at the start of fluid therapy and 24 h after and revealed an increase in the TARC levels from 689 to 3,882 pg/mL over time. Figure 1 and Table II show the course and laboratory data of cod OFC. The patient was diagnosed with cod FPIES, and her guardian was instructed to eliminate cod. Thereafter, the guardian reported that the patient consumed shark meat. OFC with 2 g of squid was performed at 5 years of age, at the guardians’ request, which caused frequent vomiting occurred. Written approval was obtained from the patient’s guardian for this case report.

## DISCUSSION

The patient met both the major (vomiting 1–4 h after food exposure and absence of skin or respiratory symptoms) and the minor (lethargy and pallor) criteria for interpretation guideline of acute FPIES OFC issued by the American Academy of Allergy, Asthma & Immunology during the cod OFC (4). In contrast to previous reports about mild to moderate symptoms in fish-induced FPIES cases (5), our findings underscore the potential for severe reactions during oral food challenges with cod. This highlights the importance of individualized management strategies in such cases. The TARC level has specifically increased and peaked 24 h after in FPIES (6). In this patient, the TARC level 24 h after symptom in our case was 3,882 pg/mL, a 5.6- to 9.1-fold increase from 422 pg/mL at the initial visit and 689 pg/mL immediately after symptom induction. The TARC level does not increase in patients with infectious gastroenteritis or immediate-type IgE-mediated food allergy (6, 7). Thus, TARC level elevation in this patient reinforces our diagnosis. ALST is a test to analyze the proliferation of peripheral blood mononuclear cells in response to antigen stimulation. According to a review (8), it could be a useful tool in the diagnosis of non-IgE-mediated gastrointestinal food allergy. However, it is important to note that ALST can also yield positive results in patients with IgE-mediated food allergies, which means that a positive ALST result alone is not sufficient to diagnose FPIES. In this case, the patient’s clinical presentation and the results of the OFC were critical in confirming the diagnosis.

The authors suspected that cod, swordfish, and squid triggered FPIES in the patient due to the multiple episodes of vomiting after ingestion. She previously experienced four vomiting episodes after ingestion of swordfish without rash and multiple episodes of urticaria after bathing. The possibility of histamine direct poisoning or nonspecific urticaria cannot be ruled out regarding urticaria during OFC with swordfish. Histamine direct poisoning is possible in histidine-rich fish, including tuna, swordfish, and mackerel (9). Swordfish is the third most prevalent cause of histamine direct poisoning in Japan (10). Thus, it seems inappropriate to diagnose her with immediate-type swordfish allergy after yielding a positive result in the OFC with swordfish. Reproducible symptoms had been identified in OFC for squid.

Sopo et al. (11) reported on fish-related FPIES and revealed that it affected 57 children who had fish-induced FPIES in Italy, of whom 35% demonstrated multiple fish intolerance and 7% had both fish- and shellfish-related FPIES. They indicated that fish FPIES are characterized by a late age of onset, long persistence, and possible tolerance to fish other than the offending fish. Of 70 patients with fish-related FPIES, 42 acquired tolerance at 4 years of age (12). A telephone survey in Japan revealed that adults with seafoods allergies consumed an average of 92.6% of seafoods species (13). This report diagnosed 18.8% of participants with FPIES. We determined the time that the patient in our case should eliminate cod, swordfish, and squid as well as the need to eliminate more food. The minimum food elimination is warranted for the patient’s quality of life even in patients with multiple food-induced FPIES.

In conclusion, FPIES is an important differential diagnosis of recurrent vomiting after ingestion of seafoods in Japanese children. Epidemiological information may be important as well as evaluation using OFC and blood tests in determining a policy. To argue this issue, more Japanese cases should be accumulated.

## Figures and Tables

**Table I:** Past laboratory data related to swordfish, cod, and squid allergy

Food triggers	Swordfish	Cod	Squid
Age at onset	2 years 4 months	3 years 2 months	3 years 2 months
Symptoms	vomit, lethargy (4 episodes)	vomit, lethargy (2 episodes)	vomit (1 episode)
Specific IgE (UA/mL)	N/A	0.15	0.1
Skin prick test	positive	negative	positive
ALST (selectivity index)	2.5	N/A	N/A
OFC	positive	positive	positive

N/A, not applicable; ALST, antigen-specific lymphocyte stimulation test.

**Table II:** Laboratory data during cod oral food challenge

	At the start of fluid therapy	24 h after symptom
White blood cell (/μL)	8,410	8,060
Neutrophil (/μL)	4,422	5,078
Hemoglobin (g/dL)	13.5	11.4
Platelet (×10^4^/μL)	28.5	23.1
CRP (mg/dL)	0.0	1.8
TARC (pg/mL)	689	3,882
Methemoglobin (%)	0.2	0.4

TARC, thymus and activation-regulated chemokine.
